# Coarse-Fine Convolutional Deep-Learning Strategy for Human Activity Recognition

**DOI:** 10.3390/s19071556

**Published:** 2019-03-31

**Authors:** Carlos Avilés-Cruz, Andrés Ferreyra-Ramírez, Arturo Zúñiga-López, Juan Villegas-Cortéz

**Affiliations:** Autonomous Metropolitan University. Electronics Department, Av. San Pablo 180, Col. Reynosa, C.P. 02200 Mexico City, Mexico; fra@azc.uam.mx (A.F.-R.); azl@azc.uam.mx (A.Z.-L.); juanvc@azc.uam.mx (J.V.-C.)

**Keywords:** CNN, deep-learning, classification, human action recognition

## Abstract

In the last decade, deep learning techniques have further improved human activity recognition (HAR) performance on several benchmark datasets. This paper presents a novel framework to classify and analyze human activities. A new convolutional neural network (CNN) strategy is applied to a single user movement recognition using a smartphone. Three parallel CNNs are used for local feature extraction, and latter they are fused in the classification task stage. The whole CNN scheme is based on a feature fusion of a fine-CNN, a medium-CNN, and a coarse-CNN. A tri-axial accelerometer and a tri-axial gyroscope sensor embedded in a smartphone are used to record the acceleration and angle signals. Six human activities successfully classified are walking, walking-upstairs, walking-downstairs, sitting, standing and laying. Performance evaluation is presented for the proposed CNN.

## 1. Introduction

Human Activity Recognition (HAR) is the automatic understanding of human actions performed by an individual or group of people. There are numerous areas and sectors where it is applied, such as smartphones, tablets, cars, games, health, security, commercial organizations and governments [1,2]. It is always been approached using sensors, namely using a video camera, infrared camera, microphone, GPS, gyroscope, accelerometer, proximity sensor, ultrasound sensor, light sensor, etc. [3,4,5,6]. Most of the sensors cited previously are integrated into a smartphone. On the other hand, in the recent years, smartphones have been preferred for implementing better HAR systems [7,8] due to the increasing accuracy of their built-in sensors, popularity, low cost, wireless facilities, and wireless connectivity. Due to the aforementioned reasons, smartphones are opening a new horizon in the applications of understanding users’ personal activities and their world contexts. In addition, the literature reports that the HAR systems embedded in smartphones are reaching good performance, however, they have not reached 100% recognition [3,4,5,6]. Single user activities that all HAR systems want to identify are walking, walking-upstairs, walking-downstairs, sitting, standing and laying, among others. The significance of the 6 activities identified in our proposed article is geared toward a future ability to assist people with disabilities in everyday household activities via a single smartphone, such as walking, ascending stairs, descending stairs, sitting, standing and laying. There are commercial HAR platforms developed by important companies such as google (https://developers.google.com/location-context/activity-recognition/), Microsoft—Azure (http://www.md2c.nl/meetup-microsoft-data-science-azure-machine-learning-workshop/), and IBM—human action recognition (https://www.ibm.com/blogs/research/category/ai/). Google “activity recognition” API identifies 2 types of movement within the 6 that we identify successfully, google HAR is interested in driving, walking, exercising, working, and playing activities. The API returns a value from 0 to 100 indicating the likelihood that the user is performing this activity. On the other hand, Microsoft—Azure API identifies sitting, standing up, standing, sitting down, walking activities, based on the use of wearable sensors. Azure API uses 4 wearing accelerometers from LiliPad Arduino positioned in the waist, left thigh, right ankle, and right arm. Azure API is not for generic use, compared to the sensors embedded in smartphones. Azure API identifies 3 types of movement within the 6 that we identify successfully. Finally, IBM—human action recognition API is oriented to HAR identification through visual information using cameras, which is beyond the scope of this article.

In this paper, a novel coarse-fine convolutional deep-learning strategy for human activity recognition is proposed which consists of three parallel CNNs that are *fine-CNN, medium-CNN,* and *coarse-CNN*. The outputs of the CNNs are flattened into a one-dimensional vector and used for the object’s classification. In this proposal, a fully-connected layer is comprised of one input layer, one hidden layer, and one output layer. Concerning the CNNs components of the proposed architecture, the *fine-CNN* consists of four feature extraction (FE) layers where each FE layer performs convolution and max-pooling tasks. Referring to the *medium-CNN*, it contains two FE layers, convolution and max-pooling each. Finally, the *coarse-CNN* has only one FE layer, applying one convolution and one max-pooling task. For all CNNs, a ReLU activation function was applied.

Because HAR research has matured, there are several public benchmark human activity datasets [3,9,10,11,12] that allow a direct comparison of different activity recognition methods. In this work, the UCI HAR dataset [3] and the WISDM dataset [13] are selected which receive signals from an accelerometer and a gyroscope smartphone sensor. Dataset recordings have six human activities: walking, walking-upstairs, walking-downstairs, sitting, standing and laying which were successfully classified throughout our proposal.

The contribution of this work is threefold. Firstly, an improved convolutional neural network for human activity recognition is implemented which successfully merges coarse, medium, and fine features information. Secondly, the parallel feature extracted in the classification layer is carried out. Finally, an improvement in the classification accuracy using a coarse-fine deep-learning strategy when compared with the related HAR works is reported.

The rest of the paper is organized as follows. Section 2 discusses the state of the art. The proposed methodology is given in Section 3. Section 4 presents the system architecture. Experiments are discussed in Section 5, followed by a comprehensive discussion on evaluation in Section 6 and Section 7. Finally, Section 8 provides discussion, conclusions and briefly clarifies the possible future direction to advance the subject.

## 2. State of the Art

In the last decade, this research area has received significant attention due to the increasing trend of HAR applications in different areas, reduction in sensor price and built-in sensors in handheld devices. The human actions are identified by applying the extraction or selection features in the time or frequency domain on the signals detected by a smartphone’s sensor. Since there are no working features that can ensure 100% identification of all the activities a person can perform, the problem still persists and requires further attention from the researchers. From sensor-based HAR research, there are two approaches.

- **Video camera sensor:** This research area is focused to identify HAR developed by a group of people. The most distinguished studies carried out for analyzing whole videos are [14,15,16,17]; 3D videos [18,19] or still images [16].
- **Infrared camera, microphone, GPS, gyroscope, accelerometer, proximity sensor, ultra-sound sensor and light sensor:** This HAR research is developed to identify a single person activity. There are survey works giving a landscape on different techniques and terminologies [14,20,21,22,23]. We are interested in this emerging research area which uses a smartphone sensor for single user recognition. The main works and their techniques are described as follows.

### 2.1. Machine Learning-Based HAR Methods

Focusing on the bibliography for a single person movement identification by a smartphone, the field of machine learning-based HAR methods reports a competitive work by Anguitia [3] which uses statistical features such as mean, minimum, maximum, standard deviation, skewness, kurtosis, angles, entropy, correlations, energy, and energy bands. The authors used support vector machine (SVM) as a classification system and they achieved good results to identify 6 human activities (walking, walking-upstairs, walking-downstairs, sitting, standing and running). The second most related work is “Human activity recognition by smartphone” by Le Tuan [4]. The author used time-domain and frequency-domain features: mean, minimum, maximum, standard deviation, energy, inter-quartile, entropy, auto-regression, correlations, skewness, kurtosis, the energy of a frequency; getting a 561-feature vector as an activity descriptor. The authors used a naive Bayes classifier and a Decision Tree criteria. Another important method is proposed in [5] were statistical features are used. By employing time-frequency features, the authors obtained good results to identify the same 6 human activities as two previous works. Other relevant work is based on Bag-of-Features [6] using a hierarchical recognition scheme over motion primitives, motion vocabulary size, weighting schemes of motion primitive assignments, and learning machine kernel functions. Also, Lane’s work [24] used a Bayesian classifier to identify 4 to 6 human activities (walking, walking-upstairs, walking-downstairs, sitting, standing and running). Other researchers [25,26,27] used a *k*-nn classifier, Kim et al. used SVM [28], Quadratic Discriminant Analysis QDA [29], Multilayer Neural Network [30], Probabilistic Neural Network [31], and Classification Rules [32].

Finally, there are works where authors applied a Hidden Markov Model for segmenting human activities [33,34], using the same public database in [3], authors obtained good results to identify the same 6 human activities considered in this research work and, they defined an “Activity sequence modeling” to identify the relationship among activities.

### 2.2. Convolutional Neural Network-Based HAR Methods

A different approach to feature extraction task is based on deep learning/CNNs, and several works have been conducted to adapt it to the HAR problem. The most related work using CNNs is [35] where authors used the “divide and conquer” paradigm and 1D convolutional neural network to identify the actions performed by humans, six activities are efficiently identified: walk, walk upstairs, walk downstairs, sit, stand, and lay. Despite the good classification, the authors did not achieve 100% accuracy. Another close work by Ignatov [36] presents a user-independent deep learning-based approach for online human activity classification. Ignatov proposes to use Convolutional Neural Networks for local feature extraction together with simple statistical features that preserve information about the global form of time series. The author investigated the impact of time series length on the recognition accuracy and limited it up to one second that makes possible the continuous real-time activity classification. The accuracy of the proposed approach is evaluated on two commonly used WISDM and UCI datasets.

Other less accurate works in HAR, using CNNs as a platform base are [37,38,39,40]. There are approaches exploiting deep Recurrent Neural Network (RNN) [41] or combined Long Short-Term Memory (LSTM) RNN with CNN. Ordonez and Roggen [42] proposed DeepConvLSTM that combined convolutional and recurrent layers. Edel et al. [43] proposed a binarized bidirectional LSTM-RNNs which reduces memory consumption and replaced most of the arithmetic operations with bitwise operations achieving an increase in power-efficiency.

Despite the variety of proposals in the HAR field using convolutional or recurrent networks, there is still an opportunity for work to achieve 100% recognition of human activities. In this paper, a novel framework is proposed to analyze and classify single user activity using a smartphone on the well-known public smartphones databases [3,13]. Our scheme is based on a coarse-fine convolutional neural network strategy which is explained in the following section.

## 3. Proposal

**Figure 1:**
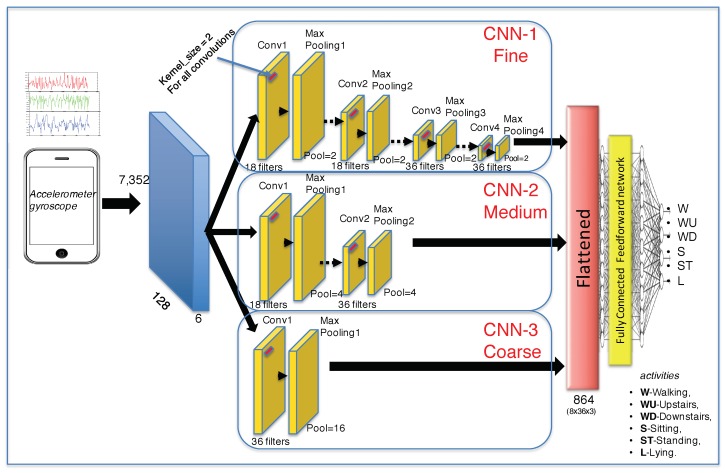
Proposed Coarse-fine convolutional neural network topology.

The architecture of the proposed coarse-fine CNN system is shown in Figure 1. CNN is a parallel feedback neural network whose structure is inspired by the visual biological system. The main idea is the hierarchization of the information visually analyzed. On one side, the “coarse” information is perceived, i.e., circles, lines, shapes, and colors. On the other side, the “average” information, and finally, the “fine” detailed information is perceived. In the present proposal, detailed information is represented by several stages of convolution and max-pooling, while “coarse” information is represented by a single stage of convolution and pooling. The three levels of information are merged in the whole classification CNN stage. The overall structure of CNNs is described below:

- *Convolutional layer:* In one-dimensional case, a convolution between two vectors $x\in R^{N}$ and a kernel vector $h\in R^{M}$ is a vector $c\in R^{M+N-1}$, where c=x∗h, * represents the convolution operation. Thus, in discrete domain, the convolution is expressed as $c\left[n\right]=\sum_{k=-\infty }^{\infty }x\left[k\right]h\left[n-k\right]$, for ∀n∈[1…N]. In other words, a reflected vector *h*, which is also called a convolutional filter, is sliding along signal *x*, a dot product is computed at each *n* value and the concatenated values c[1],c[2],…c[i] form the outputs of the convolutional layer c[n].
- *Activation function:* Among the main non-linear activation functions such as sigmoidal, tangent, hyperbolic tangent, and ReLU, the latter is used in this proposal. Rectified linear unit (ReLU) is defined as ReLU(c)=max(0,c). The effect produced by the ReLU function is thresholding of convolution *c* with respect to zero value, obtaining, only positive values of *c*.
- *Pooling layer:* The aim of this stage is to reduce and summarize the convolutional output. Two typical pooling functions are used, the max pooling and mean pooling function. In this proposal, the max pooling function is used with a vector size of [1×2].
- *Full-connected layer:* This stage concatenates the outputs of the three partial CNNs: a fine-CNN, a medium-CNN, and a coarse-CNN. The output of the partial CNNs is flattened into a one-dimensional vector and used for the classification. In this proposal, a fully-connected layer is comprised of one input layer, one hidden layer, and one output layer.
- *Soft-max layer:* Finally, the output of the last layer is passed to a soft-max layer that computes the probability distribution over the predicted walking, up-stairs, down-stairs, sitting, standing and laying human activities.

All three partial CNNs: a fine-CNN, a medium-CNN, and a coarse-CNN are trained as a whole one. Training and optimizing tasks are carried out using a back propagation algorithm and stochastic gradient descent, respectively.

## 4. System Architecture

**Figure 2:**
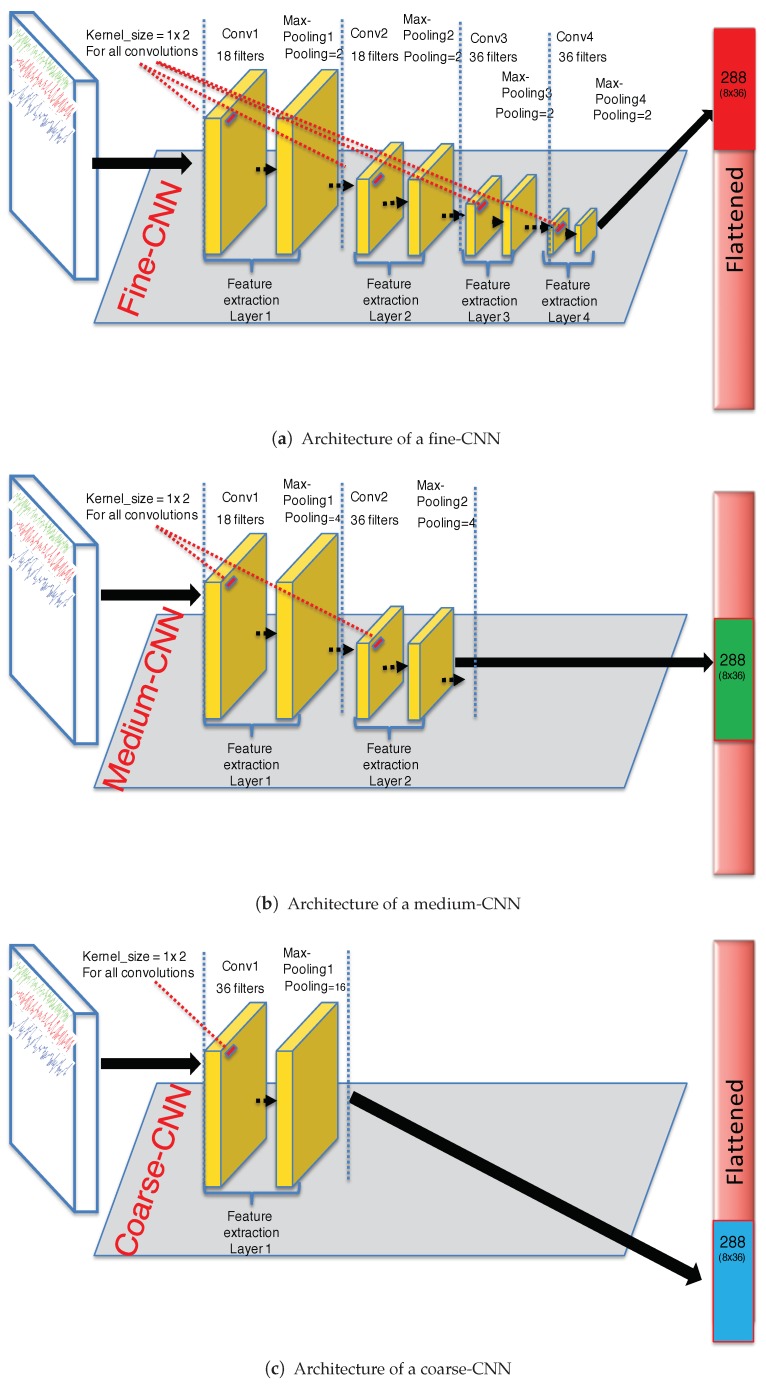
Partial CNN proposed architecture.

The whole proposed CNN architecture presented in Figure 1 is fed by six signals coming from an accelerometer and a gyroscope. The input data passes throughout the three partial CNN as follow:

- ***Fine-CNN (See Figure 2a):*** A first convolutional layer comprised of 18 filters where the kernel filter $h_{1}$ has the size 1×2 and the step of the convolution is 1. Then, a $max-pooling_{1}$ layer is applied with a size of 1×2 and the step of $max-pooling_{1}$ is 2. The activation function is ReLU. Then, a second convolutional layer, comprised of 18 filters where the kernel filter $h_{2}$ has the size 1×2 and the step of the convolution 2 is 1. Then, a $max-pooling_{2}$ layer is applied with a size of 1×2 and the step of $max-pooling_{2}$ is 2. The activation function is ReLU. A third convolutional layer comprised of 36 filters where the kernel filter $h_{3}$ has the size 1×2 and the step of the convolution 3 is 1. Then, a $max-pooling_{3}$ layer is applied with a size of 1×2 and the step of $max-pooling_{3}$ is 2. The activation function is ReLU. Finally, a fourth convolutional layer comprised of 36 filters where the kernel filter $h_{4}$ has the size 1×2 and the step of the convolution 4 is 1. Then, a $max-pooling_{4}$ layer is applied with a size of 1×2 and the step of $max-pooling_{4}$ is 2. The activation function is ReLU.
- ***Medium-CNN (See Figure 2b):*** For this CNN, first convolutional layer comprised of 18 filters where the kernel filter $h_{1}$ has the size 1×2 and the step of the convolution is 2. Then, a $max-pooling_{1}$ layer is applied with a size of 1×4 and the step of $max-pooling_{1}$ is 2. The activation function is ReLU. Then, a second convolutional layer, comprised of 36 filters where the kernel filter $h_{2}$ has the size 1×2 and the step of the convolution 2 is 3. Then, a $max-pooling_{2}$ layer is applied with a size of 1×4 and the step of $max-pooling_{2}$ is 2. The activation function is also ReLU.
- ***Coarse-CNN (See Figure 2c):*** For the last partial CNN, only one convolutional layer comprised of 36 filters where the kernel filter $h_{1}$ has the size 1×2 and the step of the convolution is 2. Then, a $max-pooling_{1}$ layer is applied with a size of 1×16 and the step of $max-pooling_{1}$ is 2. The activation function is ReLU.

The output of the three partial max-pooling output layers are then flattened. The joint vector is subsequently passed to a fully-connected layer that consists of 864 neurons (8×36×3). We have used a dropout technique in this layer with dropout rate of 0.00005. Finally, the outputs of the fully-connected layer are passed to a soft-max layer that computes probability distribution over six activity classes. The model is trained to minimize cross-entropy loss function using back propagation algorithm and optimize training parameters with stochastic gradient descent [44].

For the proposed fine-coarse CNN, the loss entropy function is defined as:

(1) $$\begin{array}{cc} \zeta_{T}\left(\Theta_{1},\Theta_{2},\Theta_{3},W\right) & =\zeta_{FMC}\left(\Theta_{1},\Theta_{2},\Theta_{3}\right)+\zeta_{CLA}\left(W\right) \end{array}$$

where $\zeta_{FMC}\left(\Theta_{1},\Theta_{2},\Theta_{3}\right)$ corresponds to the loss function of fine-CNN, medium-CNN, and coarse-CNN; and $\zeta_{CLA}\left(W\right)$ corresponds to the loss function of the whole classification layer (*dropout* and *soft-max* layers). Total loss function $\zeta_{T}$ can be rewritten as:

(2) $$\begin{array}{cc} \zeta_{T}\left(\Theta_{1},\Theta_{2},\Theta_{3},W\right) & =-\sum_{i=1}^{3}log\left[\hat{p}\left(m_{h}/\Theta_{i}\right)\right]-log\left[\hat{p}\left(m_{h}/W\right)\right]\\ & =-log\left[\hat{p}\left(m_{h}/\Theta_{1}\right)\right]-log\left[\hat{p}\left(m_{h}/\Theta_{2}\right)\right]-\\ & \;log[\hat{p}\left(m_{h}/\Theta_{3}\right)-log\left[\hat{p}\left(m_{h}/W\right)\right] \end{array}$$

where $\Omega =\{\Theta_{1},\Theta_{2},\Theta_{3}\}$ are the parameter sets for the three partial CNN. *W* is the parameter sets for the classification layer (*dropout* and *soft-max* layers). $m_{h}$ = movement type, where *h* = {walking, walking-upstairs, walking-downstairs, sitting, standing and laying}. $\hat{p}\left(m_{h}/\Theta_{i}\right)$ stands for the conditional probability function for a given movement type conditioned to a $\Theta_{i}$ parameter sets, and $\hat{p}\left(m_{h}/W\right)$ stands for the conditional probability function for a given movement type conditioned to a *W* classification parameter sets layer.

The parameter set for each partial CNN is defined as follows:

(3) $$\begin{array}{cc} \Theta_{1} & =\mathrm{parameter}\mathrm{sets}\mathrm{for}\mathrm{the}\mathrm{fine}-\mathrm{CNN}\\ \Theta_{2} & =\mathrm{parameter}\mathrm{sets}\mathrm{for}\mathrm{the}\mathrm{medium}-\mathrm{CNN}\\ \Theta_{3} & =\mathrm{parameter}\mathrm{sets}\mathrm{for}\mathrm{the}\mathrm{coarse}-\mathrm{CNN}\\ W & =\mathrm{parameter}\mathrm{sets}\mathrm{for}\mathrm{the}\mathrm{classification}\mathrm{layer}(\mathrm{dropout}\mathrm{and}\mathrm{soft}-\max\mathrm{layers}) \end{array}$$

## 5. Experiments

### 5.1. UCI HAR Dataset

**Figure 3:**
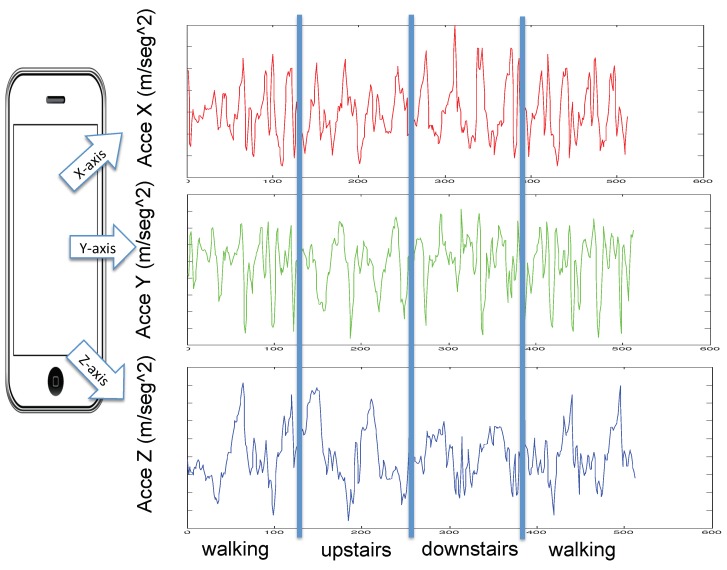
Example of four human activities: walking, walking-upstairs, and walking-downstairs.

Accelerometer and gyroscope sensors built-in in a smartphone were used to collect two-tri-axial movement information [3]. Sensor’s data were collected from 30 volunteers, between the age of 19–49 year. Carrying a smartphone Samsung Galaxy SII in a vertical position in their pockets, each subject performed six activities: walking, walking up stairs, walking down stairs, sitting, standing, and laying activity. 3-axial linear acceleration and 3-axial angular velocity data were collected. These sensor’s data were sampled at a constant rate of 50 Hz, using the embedded accelerometer and gyroscope. A realization of a single activity was divided into windows of 2.56 s each, which is sampled at 50 Hz giving 128 samples (2.56 s × 50 Hz = 128). The database is structured into two sets, 70% of the volunteers (21 persons) were selected for training and 30% for testing (9 persons). Table 1 shows the activities distribution over the two sets. An example of a single recording can be found in Figure 3 where four activities are shown. Table 2 shows the hyperparameters experimental setup. As you can see in *pooling size parameter*, there are three vector size [1×2],[1×4]and[1×16] for fine-CNN, medium-CNN and coarse-CNN, respectively (See Figure 2).

### 5.2. Data Set

1. ***Subject-Dependent dataset***
  For the training stage (See second column of Table 1), a full learning database was formed by 7352 trails of 21 volunteers (70% of the whole database). Training dataset is conformed of 7352 trials multiply by 128 samples multiply by 6 axis (7352×128×6 matrix).
2. ***Subject-Independent dataset***
  For the testing stage (See third column of Table 1), a database was formed by 2947 trails of 9 volunteers (30% of the whole database). Testing dataset is conformed of 2947 trials multiply by 128 samples multiply by 6 axis (2947×128×6 matrix).

All signals were filtered with a digital FIR low-pass filter with a cut-off frequency of 10Hz. Thus, the already filtered signals are used in the proposed neural network.

## 6. Evaluation

We implemented the proposed coarse-fine convolutional deep-learning strategy for human activity recognition on the python+Tensor Flow (python = 2.7, tensorflow = 1.1) platform running on iMac-XOS Intel Core i5 CPU. To evaluate the proposal, firstly, the influence of each partial CNN is evaluated, and then, whole parallel CNN strategy is evaluated. Performance evaluation for the three proposed CNN: Fine-CNN, Medium-CNN, and Coarse-CNN, as well as, for the proposed merged architecture is presented as follows.

### 6.1. Learning Evaluation

**Figure 4:**
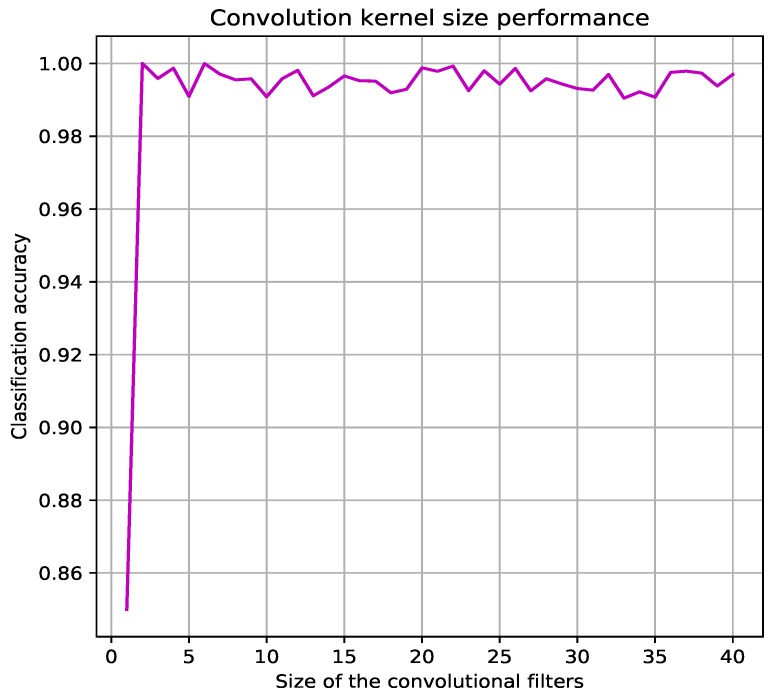
Dependency between the size of the convolutional filters and CNN accuracy.

Evaluation is developed in training and testing tasks, regarding training task, accuracy and loss parameters are evaluated for the four CNNs. One of the most important parameter to be defined is the size of the convolutional filters, which was defined experimentally to [1×2]. Figure 4 shows the classification accuracy curve, the Coarse-Fine CNN is not very sensitive to this parameter: while the first best accuracy was obtained for filter of size [1×2], the accuracy does not drop significantly till this size becomes greater than [1×2] size.

**Figure 5:**
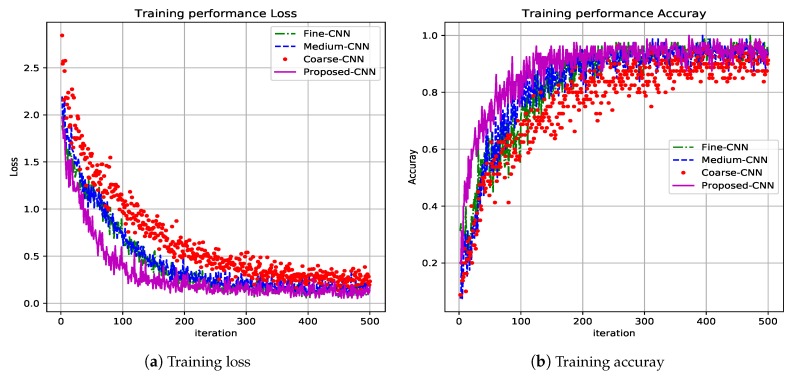
Training results for the proposed CNN architecture.

Figure 5 shows the first 500 iterations. In Figure 5a the loss function performance for the four CNN proposed: Fine-CNN, Medium-CNN, Coarse-CNN and Proposed-CNN is shown. The proposed CNN (magenta plot) reaches minimum values in less iterations and obtains zero value at the end of the training task. Besides, Figure 5b depicts the precision performance for the same four CNN, it can be seen that it is the best performance reached by the proposed CNN (magenta plot), i.e., less iterations and best accuracy.

**Figure 6:**
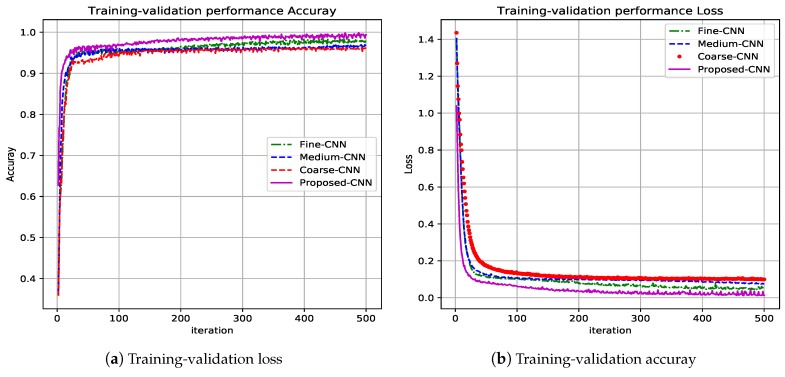
Training-validation results for the proposed CNN architecture.

Other parameters analyzed in the training task were training-validation loss and training-validation accuracy. Figure 6 shows the evolution curves through iterations. The training task is developed following the paradigm “Subject-dependent test”, it means the same dataset is used for learning and testing task. From Figure 6, it can be seen that continued magenta line plot corresponds to the proposed CNN architecture where the accuracy reaches 100% (see Figure 6b), and the loss parameter reaches the zero value (see Figure 6a). Other color plots correspond to the partial CNN used and fused: red→coarse-CNN, blue→medium-CNN, and green→fine-CNN.

### 6.2. Cross-Dataset Evaluation

**Figure 7:**
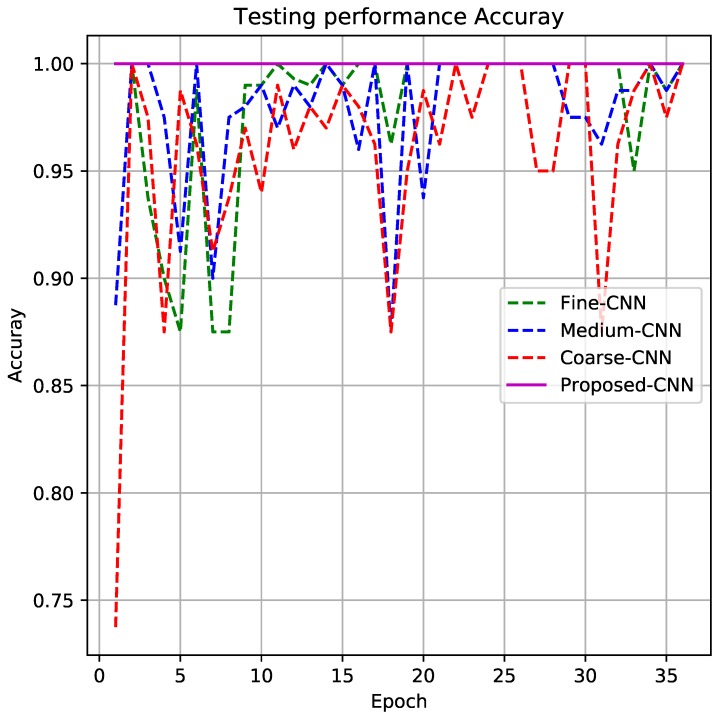
Testing results for the proposed CNN architecture.

Figure 7 shows the testing performance for the proposed CNN. For this test, the testing dataset is completely different from the learning task. The test is developed following the paradigm “Subject-Independent test”. From Figure 7, it can be seen that the continued magenta line plot corresponds to the proposed CNN architecture where the accuracy reaches 100%. It seems that the fusion of partial information given by fine-CNN, medium-CNN, and coarse-CNN makes it possible to obtain a 100% of good classification for HAR activities.

Confusion matrix of the six single user activities classification in testing task is given in Table 3, performance activities are: walking activity 100%, ascending stairs 100%, descending stairs 100%, sitting 100%, standing 100% and laying 100%; giving a mean average of 100%.

### 6.3. Comparison to Related Work

**Figure 8:**
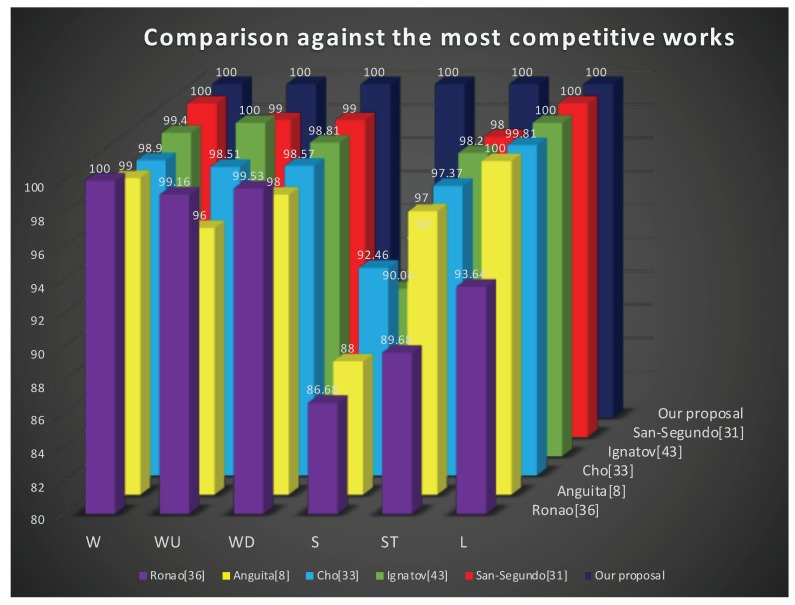
Performance comparison against the most competitive methods: W->walking, WU->walking-upstairs, WD->walking-downstairs, S->sitting, ST->standing and L->laying.

Table 4 and Figure 8 compare our coarse-fine convolutional deep-learning strategy for human activity recognition with the best competitive works reported in the literature. Please note that in this comparison, we present the classification performance for the six single user activities, as well as, the mean average performance. The comparison includes the two best competitive methods using machine learning approach [3,33], and, on the other hand, the 3 most competitive works using convolutional networks [35,36,38]. As it can be seen, our proposal accurately recognizes (100%) each of the movements under the scheme of fusion of fine, medium and coarse information from the defined convolutional neural networks.

As given in Table 4 where authors used the same database, the proposed method improves the best result from the literature about 2% i.e., from 98% San-Segundo [33] to 100%.

## 7. WISDM Dataset

To test our proposal, this paper, uses a second standard HAR dataset which is publicly available from the WISDM group [13]. The dataset is conformed of 6 activities walking, jogging, walking upstairs, walking downstairs, sitting, and standing. While performing these activities, the sampling rate for accelerometer sensor was set to 20 Hz. Dataset description is shown in Table 5.

### 7.1. Evaluation

Following the same methodology of the proposed Coarse-fine convolutional network and with the same parameters defined in Section 5 “Experiments”, the experimentation was carried out with the second WISDM dataset. The results obtained are shown in the confusion matrix where six single user activities classification in testing task is given in Table 6, performance activities are: walking activity 100%, jogging 100%, upstairs 100%, downstairs 100%, sitting 100% and standing 100%; giving a mean average of 100%.

### 7.2. Comparison to Related Work

Table 7 compares our coarse-fine convolutional deep-learning strategy for human activity recognition with the best competitive work reported in the literature. Please note that in this comparison, we present the classification performance for the six single user activities, as well as, the mean average performance. The comparison is versus the most competitive work using convolutional networks [45]. As it can be seen, our proposal accurately recognizes (100%) each of the movements under the scheme of fusion of fine, medium and coarse information from the defined convolutional neural networks.

As given in Table 7 where the author used the same database and a CNN, the proposed method improves about 0.7% i.e., from 99.33% to 100%.

## 8. Conclusions

Human activity recognition is a challenging problem. In this paper, a novel CNN framework is presented to classify single user activities based on local feature extraction under parallel scheme. The whole CNN strategy is based on coarse-medium-fine feature extraction and then, their fusion in a classification stage.

The sensors used to record the acceleration and angle signals were a tri-axial accelerometer and a tri-axial gyroscope embedded in a smartphone.

Six human activities were successfully classified: walking, walking-upstairs, walking-downstairs, sitting, standing and laying, giving an average recognition of 100%.

The experimental results show that our proposal outperforms the most competitive methods reported in San-Segundo [33], Ignatov [36], Cho [35], Anguita D. [3], Ronao [38], and [45]; with an average recognition of 100% for the same human activity.

Future work includes taking into account the more complex human activities and to find association relationships to health issues for common physical diseases. 

## Figures and Tables

**Table 1:** Activities distribution over the training and testing sets in UCI HAR database.

	Training	Testing
walking	1226	496
walking-upstairs	1073	471
walking-downstairs	986	420
sitting	1286	491
standing	1374	532
laying	1407	537
*Total*	7352	2947

**Table 2:** Experiment setup.

Parameter	Value
The size of input vector	128
The number of input channels	6
Filter size	[1×2]
Pooling size	[1×2],[1×4]or[1×16]
Activation function	ReLU (rectified linear unit)
Learning rate	0.0001
Weight decay	0.00005
Momentum	0.5–0.99
The probability of dropout	0.8
The size of minibatches	500
Maximum epochs	2000

**Table 3:** Testing confusion matrix of the six single user activities.

	Walking	Ascending Stairs	Descending Stairs	Sitting	Standing	Laying
walking	100	0	0	0	0	0
ascending stairs	0	100	0	0	0	0
descending stairs	0	0	100	0	0	0
sitting	0	0	0	100	0	0
standing	0	0	0	0	100	0
laying	0	0	0	0	0	100

**Table 4:** Comparison performance of the most competitive methods in detection of human motion activities recognition using the same database.

	Walking	Ascending Stairs	Descending Stairs	Sitting	Standing	Laying	Mean Average
**Our proposal**	100%	100%	100%	100%	100%	100%	100%
San-Segundo [33]	100%	99%	99%	92%	98%	100%	98%
Ignatov [36]	99.4%	100%	98.81%	90.04%	98.2%	100%	97.63%
Cho [35]	98.9%	98.51%	98.57%	92.46%	97.37%	99.81%	97.62%
Anguita [3]	99%	96%	98%	88%	97%	100	96%
Ronao [38]	100%	99.16%	99.53%	86.68%	89.69%	93.64	94.79%

**Table 5:** WISDM dataset description [13].

Activity	Number of Samples	Percentage
*Walking*	424,400	38.6%
*Jogging*	342,177	31.2%
*Upstairs*	122,869	11.2%
*Downstairs*	100,427	9.1%
*Sitting*	59,939	5.5%
*Standing*	48,397	4.4%

**Table 6:** Testing confusion matrix of the six single user activities.

	Walking	Jogging	Upstairs	Downstairs	Sitting	Standing
walking	100	0	0	0	0	0
jogging	0	100	0	0	0	0
upstairs	0	0	100	0	0	0
downstairs	0	0	0	100	0	0
sitting	0	0	0	0	100	0
standing	0	0	0	0	0	100

**Table 7:** Comparison performance of the most competitive method in detection of human motion activities recognition using WISDM dataset.

	Walking	Jogging	Upstairs	Downstairs	Sitting	Standing	Mean Average
**Our proposal**	100%	100%	100%	100%	100%	100%	100%
Shakya [45]	98%	99%	100%	99%	100%	100%	99.3%
